# Use of the myocutaneous serratus anterior free flap for reconstruction after salvage glossectomy

**DOI:** 10.1007/s00405-018-5245-4

**Published:** 2018-12-14

**Authors:** Stefan Janik, Julian Pyka, Isabella Stanisz, Tamara Wachholbinger, Matthias Leonhard, Imme Roesner, Doris-Maria Denk-Linnert, Brett A. Miles, Berit Schneider-Stickler, Boban M. Erovic

**Affiliations:** 1 Department of Otolaryngology, Head and Neck Surgery, Medical University of Vienna, Vienna, Austria; 2 Division of Phoniatrics-Logopedics, Department of Otolaryngology, Medical University of Vienna, Vienna, Austria; 3 Department of Otolaryngology Head and Neck Surgery, Icahn School of Medicine at Mount Sinai, New York, NY USA; 4 Institute of Head and Neck Diseases, Evangelical Hospital Vienna, Vienna, Austria

**Keywords:** Myocutaneous SAFF, Salvage glossectomy, Subtotal glossectomy, Total glossectomy, Functional outcomes

## Abstract

**Purpose:**

To describe the use of a myocutaneous serratus anterior free flap (SAFF) for tongue reconstruction after salvage subtotal (STG) and total glossectomy (TG).

**Methods:**

In this prospective case series, seven patients underwent salvage STG or TG and reconstruction with a myocutaneous SAFF between 10/2015 and 02/2017. Functional and oncologic outcomes were prospectively evaluated. Donor side morbidity was determined using the Disabilities of the Arm, Shoulder and Hand (DASH) score.

**Results:**

SAFF with mean skin paddles of 6.7 cm × 8.7 cm was used in five STG and two TG patients, respectively. There was a 100% flap survival and a mean DASH score of 10.8 reflected normal arm and shoulder function after surgery. One year after salvage surgery, 1 (14.3%) and 4 (57.1%) patients were tracheostomy and gastrostomy tube dependent. Gastrostomy tube dependence was significantly worse in patients with tumors of the base of tongue compared to other tumor sites (*p* = 0.030) and in patients who underwent transcervical compared to transoral tumor resection (*p* = 0.008). Local recurrence rate was 57.1% with a disease-free survival of 17.6 months.

**Conclusion:**

The myocutaneous SAFF represents a safe and reliable flap for tongue reconstruction after salvage glossectomy with satisfying functional outcomes and low donor side morbidity.

## Introduction

Subtotal glossectomy (STG) and total glossectomy (TG) are surgical options in cases of advanced stage or recurrent carcinomas originating from the floor of mouth (FOM), the oral part of the tongue (OT) or base of the tongue (BOT) [1]. Despite necessary extensive tumor resections and adjuvant treatment regimens, poor 3- and 5-year disease-specific survival (DSS) rates of 38–51% and 25–41% are reported, respectively [2–4].

Tongue reconstruction remains one of the most challenging problems in head and neck surgery [1–5]. Successful tongue reconstruction includes more than satisfying wound healing, wound closure and survival of free flaps [5]. Within the past years, an increasing number of studies has focused on functional outcomes after reconstruction, such as sufficient oral nutrition and functional, intelligible speech, and how to optimize these functional outcomes [5, 6].

The aim of tongue reconstruction differs depending on whether the OT or the BOT is affected. Principally, the OT is essential for speech, mastication, oral hygiene, the oral and oropharyngeal phase of swallowing [7]. Therefore, thin, pliable flaps, such as the radial forearm free flap (RFFF), are commonly used [5].

Otherwise, the BOT is crucial for completing the pharyngeal phase of swallowing and for prevention of aspiration [8]. Subsequently, large, bulky free flaps, such as the anterolateral thigh (ALT) or the rectus abdominis (RA) flap, are necessary to enable oral bolus propulsion into the pharynx and to assist speech articulation [1, 5, 9].

Numerous studies have already evaluated the impact of free flap reconstruction after glossectomy on oncologic and functional outcomes [9–14]. However, no studies have reported on the use of the myocutaneous serratus anterior free flap (SAFF) for tongue reconstruction after STG and TG. The reliable vascular supply and the branching pattern of the associated vasculature of the SAFF, allow harvesting of up to five muscle digitations with skin islands up to 20 × 20 cm of the same vascular pedicle [15, 16].

Therefore, the myocutaneous SAFF represents a suitable candidate for tongue rehabilitation that provides the needed versatility for reconstruction of the OT and BOT with one free flap. Hence, it was the main purpose of this study to evaluate the suitability of the myocutaneous SAFF for tongue reconstruction and to evaluate oncologic and functional outcomes.

## Material and methods

### Study cohort

A prospective case series was conducted on seven patients who underwent salvage STG and TG with laryngeal preservation, and reconstruction with myocutaneous SAFF at the Department of Otolaryngology, Head and Neck Surgery, Medical University of Vienna. Surgical resectability, adequate general health status, and the necessity of subtotal or total salvage glossectomy with free flap reconstruction were considered as inclusion criteria for this study. Conversely, patients undergoing partial or hemiglossectomy, regardless of free flap reconstruction, were excluded from this analysis. Tumor resections and reconstructions were performed by the same head and neck surgeon (B.E.) between 10/2015 and 02/2017. Similar to former studies, we defined TG as a removal of the entire tongue and STG as resection of more than two-thirds of the oral part of the tongue with preservation of the BOT [1].

All patients were initially staged by clinical examination, computed tomography scans or magnetic resonance imaging scans of the head and neck followed by chest imaging. Data were collected regarding patient age, sex, former therapies, histopathologic results, pre- and postoperative therapies, oncologic outcome parameters, including disease-specific survival (DSS) and disease-free survival (DFS), and functional outcomes.

### Functional outcomes

Swallowing, gastrostomy tube dependence, and tracheostomy dependence were evaluated as functional parameters. Functional outcomes were assessed preoperatively (baseline check-up), 2 weeks (1st check-up), 4 weeks (2nd check-up), 6 months (3rd check-up) and 1 year postoperatively (final check-up). Fiberoptic endoscopic evaluation of swallowing (FEES) was performed by experienced phoniatricians (B.S.S., M.L., I.R., D.M.-D.L.) to assess swallowing function at the five check-ups. Nasogastric feeding or gastrostomy tube were removed, if patients were able to achieve adequate oral nutrition without aspiration.

### DASH questionnaire

The Disabilities of the Arm, Shoulder and Hand (DASH) questionnaire was developed by the American Academy of Orthopedic Surgeons to evaluate upper limb-related activities, participation, symptoms and disabilities [17]. Its reliability and validity for a variety of upper limb disorders have been already shown [18–20]. The DASH questionnaire consists of 30 items, and a five-point Likert scale (1–5) is used to evaluate 21 items regarding daily activities, 5 regarding symptoms, 3 regarding participation and impact on daily life and 1 item about confidence in abilities (0 = no disability; 100 = most severe disability). According to Hunsaker et al. [21], who evaluated normative values of general US population for musculoskeletal outcome measures, a DASH score of 10.1 is assumed as healthy norm. Furthermore, DASH scores between 0 and 15, 16 and 45 and 46 and 100 are classified as “normal or minimal upper-limb disability”, as “mild disability but still able to work” and as “severe upper-limb disability that prevents from working”, respectively. The DASH questionnaire was accomplished 6 months after salvage surgery to assess the impact of SAFF harvest on shoulder and arm function.

### Statistical methods

Statistical analyses were performed using SPSS software (version 21; IBM SPSS Inc., IL, USA). We mainly performed descriptive analysis. All data are indicated as mean ± standard deviation (SD) in “Results”. Unpaired Student’s *t* test was used to compare means of normally distributed variables, while Chi-square test was performed to investigate the association between nominal variables. All tests were two-sided and *p* values below 0.05 were considered as statistically significant.

## Results

### Patient cohort

We included seven patients (6 male, 1 female) with a mean age of 54.7 ± 8.6 years (range 38.4–66.9 years) who underwent salvage glossectomy. Primary radiochemotherapy (pRChT), partial glossectomy with adjuvant radiotherapy (RT) and partial glossectomy with adjuvant RChT were accomplished in 2 (28.6%), 2 (28.6%) and 3 patients (42.8%) as primary treatment regimens, respectively. Standard dosage of RT was 66–70 Gy and platinum-based chemotherapy regimens were used in those five patients, who received RChT. The mean time between primary therapy and salvage glossectomy was 19.1 months with a range of 4–60 months. Two patients had residual tongue carcinomas and the remaining five patients had local recurrences (Table 1).

**Table 1:** Patient, disease and treatment characteristics

Case	Sex	Age (years)	Primary therapy	Time to salvage surgery (months)	Residuum vs. recurrence	Tumor stage	Origin	Approach and extent of surgery	
I	M	55.3	RChT	36	Recurrence	T3 N0	OT	Oral	STG
II	M	58.8	Surgery RChT	16	Recurrence	T4 N0	FOM	Cervical	STG
III	F	66.9	Surgery RChT	60	Recurrence	T2 N0	BOT	Cervical	TG
IV	M	62.2	RChT	4	Residuum	T3 N1	OT	Oral	STG
V	M	51.1	Surgery RT	6	Recurrence	T3 N0	BOT	Cervical	STG
VI	M	38.4	Surgery RChT	9	Residuum	T3 N1	BOT	Cervical	TG
VII	M	50.3	Surgery RT	3	Recurrence	T3 N0	OT	Oral	STG

*M* male, *F* female, *RChT* radiochemotherapy, *RT* radiotherapy, *OT* oral tongue, *FOM* floor of mouth, *BOT* base of tongue, *STG* subtotal glossectomy, *TG* total glossectomy

### Tumor localization, extent of surgery and reconstruction with SAFF

Tumors originated mainly from OT, BOT and FOM in three (42.9%), three (42.9%) and one (14.3%) cases, respectively. We had five T3 (71.4%), one T4 (14.3%) and one T2 (14.3%) moderately differentiated (G2) squamous cell carcinoma (SCC), respectively. Positive neck nodes were found in two out of seven patients (28.6%). STG and TG were performed in five (71.4%) and two (28.6%) patients, respectively. Transcervical visor approach (*n* = 4) was used for those two patients who underwent TG and in two patients with STG due to trismus to improve exposure. The remaining three patients who underwent STG received tumor resection via an oral approach (Table 1). Radical tumor resection (R0) with negative surgical margins (> 5 mm safety margin) were confirmed in all cases by final pathology.

**Fig. 1:**
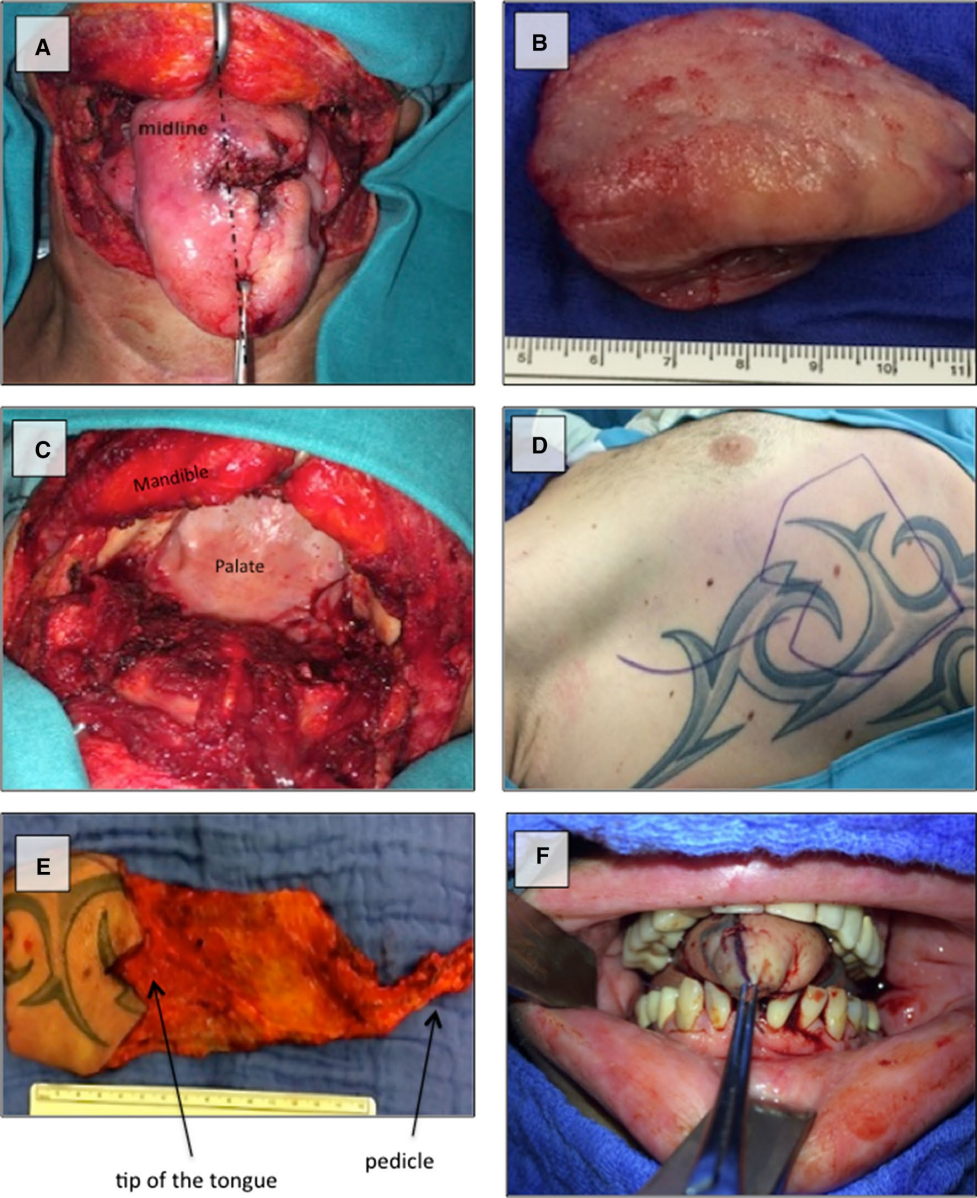
Total glossectomy via transcervical approach and reconstruction. A 38-year-old male patient with recurrent squamous cell carcinoma, originating from the left base of the tongue trespassing the midline to the right (**a**), underwent total glossectomy (**b**). Transcervical approach was performed for tumor resection (**c**). Reconstruction was done with a right-sided myocutaneous serratus anterior free flap (size of skin island 10 × 10 cm) (**d, e**). Final, reconstructed neo-tongue with adapted tip of the tongue (**f**)

Right-sided myocutaneous SAFF with the three lowest digitations was used in all patients for tongue reconstruction (Fig. 1). Mean size of skin islands was 6.7 × 8.7 cm (range 4–10 cm × 7–11 cm). All patients had uneventful postoperative courses with 100% flap survival.

### DASH score

In addition to flap survival, the DASH score was conducted to evaluate how flap harvest impacts upper limb function and activities of daily life. DASH scores of our cohort were additionally compared to the scores of the healthy norm, which was found to be 10.1 by Hunsaker et al. [21]. All patients completed the DASH questionnaire 6 months after surgery. Mean and median DASH scores were 10.8 and 5.0 (range 0.9–40.7), respectively. Mean DASH score of our cohort was not significantly different compared to healthy norm (10.8 vs. 10.1; *p* = 0.967). Moreover, DASH score did not significantly correlate with age (*p* = 0.368), sex (*p* = 0.721), extent of salvage glossectomy (*p* = 0.431), surgical approach (*p* = 0.772) or size of harvested skin island of SAFF (*p* = 0.536).

### Functional outcomes: tracheostomy and gastrostomy tube dependence

Surgical tracheostomy with creation of a Björk flap was performed in all patients during tumor resection. Successful decannulation was achieved in 4 (57.1%), 6 (85.7%) and 6 (85.7%) patients 1, 6 and 12 months after salvage glossectomy with free flap reconstruction, respectively.

FEES was performed within check-ups according to the five defined time points. Sufficient oral intake without a gastrostomy tube (PEG) was possible in six patients (85.7%) before salvage glossectomy. One female patient, who had previously undergone hemimaxillectomy, needed a PEG to maintain her caloric requirement. Principally, all patients received a nasogastric feeding tube at the end of the tumor surgery, except the female patient with preoperative PEG. One month after salvage surgery (2nd check-up), four patients with nasogastric feeding tube did not maintain their caloric requirement and, therefore, PEG tube placement was necessary. Because of logopedic dysphagia therapy, PEG tube could be removed in one patient between the second and third check-ups. Accordingly, 5 (71.4%), 4 (57.1%) and 4 (57.1%) patients were PEG dependent 1, 6 and 12 months after surgery (Table 2). Missing oral bolus transport, decreased tilting of the epiglottis and silent aspiration were the main problems for PEG tube dependence in our cohort. At 12 months after salvage glossectomy, there was a trend towards increased dependence on a gastrostomy tube in patients who underwent TG compared to STG, which did not reach statistical significance (*p* = 0.147). Moreover, regardless whether TG or STG was performed, all patients (4 out of 4) who underwent salvage glossectomy via transcervical approach remained totally PEG dependent, while all patients who had undergone oral tumor resection (3 out of 3) showed sufficient oral intake (*p* = 0.008). Additionally, patients with tumors originating from BOT were significantly more often PEG dependent (*p* = 0.047) (Table 3).

**Table 2:** Functional outcomes

Case	Tracheostomy dependence					Gastrostomy tube dependence				
	Base	1st	2nd	3rd	Final	Base	1st	2nd	3rd	Final
I	−	+	+	−	−	−	−	+	−	−
II	−	+	+	+	+	−	−	+	+	+
III	−	+	+	−	−	+	+	+	+	+
IV	−	+	−	−	−	−	−	−	−	−
V	−	+	−	−	−	−	−	+	+	+
VI	−	+	−	−	−	−	−	+	+	+
VII	−	+	−	−	−	−	−	−	−	−
Total (%)	0/7 (0)	7/7 (100)	3/7 (42.9)	1/7 (14.3)	1/7 (14.3)	1/7 (14.3)	1/7 (14.3)	5/7 (71.4)	4/7 (57.1)	4/7 (57.1)

Tracheostomy and gastrostomy tube dependence (PEG) are shown for our patient cohort according to the five check-ups. Plus (+) indicates dependence, while minus (−) indicates independence

**Table 3:** Gastrostomy tube dependence according to different surgical characteristics

Gastrostomy tube dependence		
Surgical characteristics	Nr. (%)	*p* value
Surgical approach		
Transoral (*n* = 3)	0 (0)	
Transcervical (*n* = 4)	4 (100)	0.008^a^
Extent of glossectomy		
Subtotal glossectomy (*n* = 5)	2 (40)	
Total glossectomy (*n* = 2)	2 (100)	0.147^a^
Tumor origin		
Oral tongue (*n* = 3)	0 (0)	
Floor of mouth (*n* = 1)	1 (100)	
Base of tongue (*n* = 3)	3 (100)	0.030^a^

Gastrostomy tube dependence 1 year after salvage glossectomy is shown according to surgical approach, extent of glossectomy and tumor origin

^a^Chi-square test

### Recurrence and survival

The length of follow-up ranged from 14.4 to 30.6 months with a mean follow-up of 19.5 ± 6.7 months. One- and 2-year DSS was 100% and 55.6%, compared to 1- and 2-year DFS of 57.1% and 57.1%, respectively.

Four patients (57.1%) experienced recurrence after salvage glossectomy with a mean time to recurrence of 17.6 months. Three of these four patients had local recurrence, while one patient developed regional recurrence. The latter experienced supraglottic recurrence 28 months after STG. Mean age of patients with recurrent disease was 50.0 ± 4.9 years, which was younger but not significantly different compared to 60.3 ± 3.4 years in those patients without recurrence (*p* = 0.187).

## Discussion

Due to its versatility, ease of harvest, low donor site morbidity, and reliable vascular supply, the SAFF has found many applications in head and neck reconstruction [16]. Nonetheless, the use of the myocutaneous SAFF for tongue reconstruction after salvage glossectomy has not been previously evaluated in the literature.

Numerous studies have already been published reporting on functional outcomes in patients undergoing glossectomy. Until now, the RFFF was preferred for reconstruction of the tip of the tongue because of its thinness and pliability, while the ALT flap was predominantly used as large and bulky flap for large tongue defects [10]. Within this prospective study, we could show that the myocutaneous SAFF represents an additional option for tongue reconstruction after salvage STG and TG. Demographic characteristics of our case series were comparable to the literature [1, 9, 12, 22].

There are several reasons why we believe that the myocutaneous SAFF is an excellent flap for tongue reconstruction that could be equal or even superior to RFFF and ALT. First, it is noteworthy that the SAFF can be harvested with up to five muscle digitations, providing enormous flexibility for reconstruction [15, 16]. Because of this versatility, it is possible to modify the bulk of the flap as required, ranging from a thin and pliable flap, comparable to a RFFF, to a large and bulky flap, similar to an ALT flap. Empirically, the bulk of the RFFF is frequently too small, while an ALT flap is often too large and, therefore, the SAFF represents a reliable alternative.

Second, there were no significant donor site morbidities and a 100% free flap survival. We used the DASH questionnaire, which evaluates upper limb disabilities, to assess donor side morbidity of free flap harvest. Mean and median DASH scores were 10.8 and 5.0 in our cohort, respectively, which are similar to mean and median DASH scores of 10.4 and 8.0 reported by Miles and Gilbert [23]. Generally, a mean DASH score of 10.1 was set as norm for representative US population [21]. Accordingly, harvest of SAFF does not have significant handicaps on upper limb function of patients compared to healthy controls. This finding is in striking contrast with the known donor site morbidity associated with the radial forearm and rectus free flaps, and certainly comparable to the anterior lateral thigh free flap, with the notable advantage of early ambulation which is beneficial in an older at risk population.

Moreover, the SAFF derives its vascular supply from the thoracodorsal artery, a branch of the subscapular and axillary artery. This central position in the vascular system provides a relative protection from peripheral vascular disease [16]. Hence, free flaps of the thoracodorsal system are particularly useful in patients with peripheral vascular disease, which could comprise the use of lower limb free flaps [24].

Though some case series reported of improved flap sensibility, speech and swallowing recovery of neurotized free flaps, systematic reviews failed to demonstrate that sensory reinnervation of neo-tongues is associated with benefits regarding functional outcomes [10, 25]. However, SAFF can be also harvested with a long thoracic nerve for potential sensory reinnervation of neo-tongue or for anastomosis with hypoglossal nerve if the surgeon feels this is warranted.

Regarding functional outcomes, 57.1% of patients remained gastrostomy tube dependent 12 months after salvage surgery, which is comparable to the literature, where gastrostomy tube dependence rates of 24–75% are reported [12–14]. Further, successful decannulation rates of 84–100% were reported in patients undergoing TG with laryngeal preservation, which equals the decannulation rate of 85.7% in our patient cohort [9, 12, 13, 26]. Altogether, there were no significant differences regarding functional outcomes, in our case series compared with other free flaps reported in previous studies.

However, we found a significantly higher rate of gastrostomy tube dependence in those patients with SCCs of the BOT and in patients who were operated via transcervical approach. It is already known that a greater extent of glossectomy results in a poorer functional outcome [5]. Particularly, quality of life regarding speech and swallowing function correlates with the extent of tongue base resection [27]. The following reasons may be at least a partial explanation for the significantly worse swallowing function after transcervical salvage glossectomy and resection of BOT. First, it was postulated that damaging or cutting the muscles of the FOM could result in poor swallowing function due to impaired, uncoordinated elevation of the hyoid and the larynx [28, 29]. Second, since we knew that large, bulky neo-tongues assist in oral bolus protrusion and completion of the pharyngeal phase of swallowing, it seems logical that preservation of a functional BOT should have a more favorable prognosis than resection and reconstruction [5, 26]. Third, significantly worse dysphagia in patients after transcervical tumor resection might be also caused by selection bias. Patients with larger carcinomas causing trismus due to tumor infiltration or fibrosis of the pterygoid muscles, which makes oral tumor resection impossible, have to undergo more invasive transcervical tumor resection. Accordingly, worse swallowing function after transcervical tumor resection can either result from surgical approach or by already preoperatively existing impaired swallowing function due to tumor extent.

Despite radical tumor resection and successful reconstruction, the oncologic outcome is principally poor in patients suffering from tongue carcinomas with tumor recurrences in 50–58% of patients. Recurrences mainly occur within the first 2 years of follow-up and the majority of these patients died of recurrent disease [9, 30, 31]. Similarly, we observed tumor recurrences in four patients (57.1%) occurring with an average time to recurrence of 17.6 months. Among those four patients, two died of extensive local recurrence within the first 2 years after glossectomy.

Overall 2-year DSS was 55.6% in our cohort, which was remarkably worse in younger adults. Popovtzer et al. [31] already reported on this extraordinary tumor behavior in young adults. Accordingly, he described two different tumor types. On the one hand, there are patients with aggressive tumor behavior and 40% mortality within the first 2 years after surgery, and on the other hand, there are patients with almost indolent course and freedom from recurrence for 20 years [31].

Although we have demonstrated for the first time that myocutaneous SAFF can be successfully used for tongue reconstruction after salvage glossectomy, there are limitations with our study. The main limitation is the small sample size of our case series and that patients with TG and STG were included and compared. This is a reflection of the low incidence of patients with extensive and resectable tongue carcinoma who are surgical candidates. Moreover, T3 and T4 tongue carcinomas are characterized by high recurrence rates and poor outcomes, as already discussed by numerous studies [2–4]. Due to these issues, robust data regarding long-term outcomes in this population remain elusive. Therefore, in our study we could provide only 12 months of follow-up of oncologic and functional outcomes, which is another limitation of the study.

## Conclusion

In conclusion, the myocutaneous SAFF represents an excellent option for free flap reconstruction after salvage STG and TG with low donor side morbidity, high rate of free flap survival, satisfying functional outcomes and extraordinary versatility.
